# Fast gene transfer into the adult zebrafish brain by herpes simplex virus 1 (HSV-1) and electroporation: methods and optogenetic applications

**DOI:** 10.3389/fncir.2014.00041

**Published:** 2014-05-06

**Authors:** Ming Zou, Paul De Koninck, Rachael L. Neve, Rainer W. Friedrich

**Affiliations:** ^1^Friedrich Miescher Institute for Biomedical ResearchBasel, Switzerland; ^2^University of BaselBasel, Switzerland; ^3^Institut Universitaire en Santé Mentale de QuébecQuébec, QC, Canada; ^4^Département de Biochimie, Microbiologie et Bio-informatique, Université LavalQuébec, QC, Canada; ^5^McGovern Institute for Brain Research, Massachusetts Institute of TechnologyCambridge, MA, USA

**Keywords:** zebrafish, adult brain, gene transfer, herpes simplex virus type I, electroporation, optogenetics, genetically encoded calcium indicator

## Abstract

The zebrafish has various advantages as a model organism to analyze the structure and function of neural circuits but efficient viruses or other tools for fast gene transfer are lacking. We show that transgenes can be introduced directly into the adult zebrafish brain by herpes simplex type I viruses (HSV-1) or electroporation. We developed a new procedure to target electroporation to defined brain areas and identified promoters that produced strong long-term expression. The fast workflow of electroporation was exploited to express multiple channelrhodopsin-2 variants and genetically encoded calcium indicators in telencephalic neurons for measurements of neuronal activity and synaptic connectivity. The results demonstrate that HSV-1 and targeted electroporation are efficient tools for gene delivery into the zebrafish brain, similar to adeno-associated viruses and lentiviruses in other species. These methods fill an important gap in the spectrum of molecular tools for zebrafish and are likely to have a wide range of applications.

## Introduction

The zebrafish is an attractive vertebrate model to analyze the structure and function of neural circuits because it is small, transparent at early developmental stages, genetically modifiable, and amenable to electrophysiological and optical measurements of neuronal activity (Friedrich et al., 2010, 2013; Leung et al., 2013). However, zebrafish do not offer efficient methods for fast neuronal gene transfer *in vivo* at post-embryonic stages. In rodents and other vertebrates, gene transfer in the brain is often accomplished by the injection of viral vectors, particularly adeno-associated viruses (AAVs) or lentiviruses (Luo et al., 2008). These vectors allow for the rapid expression of transgenes in spatially defined brain areas and can be targeted to defined subsets of cells by specific promoters and intersectional genetic approaches. As a consequence, viral gene transfer has become an important tool for a wide range of applications including optical measurements and manipulations of neuronal activity using genetically encoded calcium indicators (GECIs) and optogenetic probes, respectively (Knöpfel et al., 2010; Yizhar et al., 2011; Pérez Koldenkova and Nagai, 2013). In zebrafish, however, commonly used AAVs or lentiviruses failed to produce detectable expression of transgenes in the brain (Zhu et al., 2009). Fast, flexible and cost-effective methods are therefore desired to express transgenes in zebrafish without the need for time-consuming production of stable transgenic lines. Here we explored other viral vectors and non-viral methods to achieve fast, robust and long-term expression of transgenes in the zebrafish brain.

Viral gene transfer in zebrafish has been achieved using baculoviruses, Rabies virus, and Sindbis virus (Wagle and Jesuthasan, 2003; Wagle et al., 2004; Zhu et al., 2009). However, these vectors have practical disadvantages including toxicity (Sindbis), complex procedures for virus production and modification (Rabies, baculoviruses), and the difficulty to produce high titers (Rabies). One possibility to circumvent these problems is to use pseudotyped letiviruses or murine leukemia viruses (Rothenaigner et al., 2011). Another class of viral vectors with favorable properties are modified herpes simplex viruses 1 (HSV-1) (Luo et al., 2008). Although HSV-1 can infect zebrafish (Burgos et al., 2008), HSV-1-derived vectors have, to our knowledge, not yet been explored as tools to introduce transgenes into zebrafish neurons.

An alternative approach for fast gene transfer is electroporation, which uses brief electrical pulses to transiently permeabilize the plasma membrane and transfer nucleic acids into cells (De Vry et al., 2010). This method does not require the production of specialized vectors, is cost-effective, and has additional advantages (Barnabé-Heider et al., 2008). Electroporation is a popular method to manipulate neurons during development (“*in utero* electroporation”) (Tabata and Nakajima, 2001) and has been used in various species (Barnabé-Heider et al., 2008; De Vry et al., 2010) including zebrafish (Rambabu et al., 2005; Cerda et al., 2006; Hendricks and Jesuthasan, 2007; Bianco et al., 2008). However, despite promising reports (Nishi et al., 1996; Rambabu et al., 2005; Barnabé-Heider et al., 2008), electroporation is not a common method to introduce transgenes directly into spatially restricted neuronal populations in the adult brain.

We found that HSV-1-derived vectors and electroporation can be used to transfer transgenes into spatially restricted populations of neurons in the adult zebrafish brain with high efficiency. Using these approaches to express different ChR2 variants and GECIs, we explored the potential of optogenetic approaches to analyze functional synaptic connectivity among sparsely connected neurons in the posterior zone of the dorsal telencephalon (Dp), the teleost homolog of olfactory cortex.

## Materials and methods

### Animals and handling for surgical procedures

Experiments were performed in wild-type zebrafish (Danio rerio) of both sexes that were raised at 25–28°C on a 14/10 h on/off light cycle. Adult fish were > 3 months old. All experimental protocols were approved by the Veterinary Department of the Canton Basel-Stadt (Switzerland).

**Figure 1:**
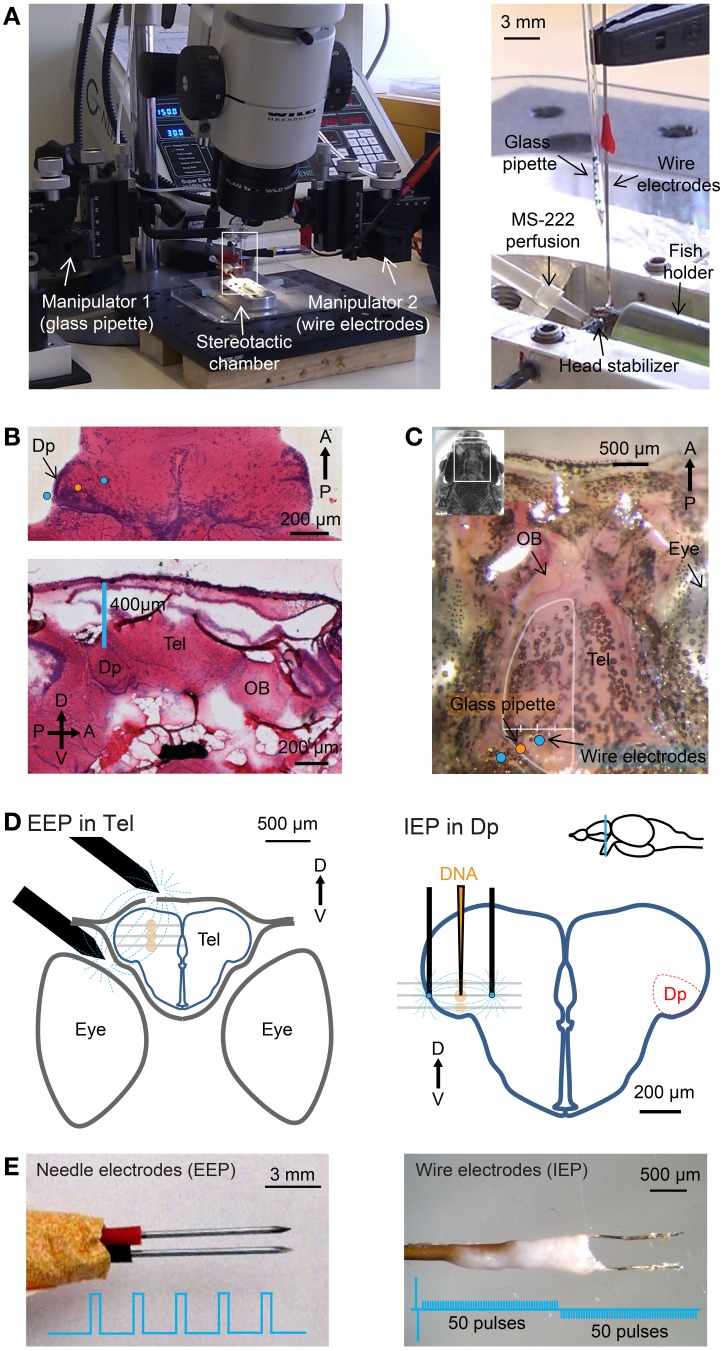
**Stereotactic injection and electroporation. (A)** Left: apparatus for injection and electroporation. Right: Arrangement of wire electrodes and glass micropipette for targeted electroporation with internal electrodes (IEP) in the stereotactic chamber. Positions of electrodes and micropipette relative to Dp are shown schematically in **(D)**. **(B)** Top: hematoxylin and eosin (H&E) staining of a horizontal brain section through Dp. Somata are stained blue. Approximate positions of injection pipette and wire electrodes for targeted IEP in Dp are indicated. Bottom: sagittal section. D, dorsal; V, ventral; A, anterior; P, posterior. **(C)** Dorsal view of the skull over the telencephalon (Tel) and olfactory bulb (OB). The bone over the left olfactory bulb has been removed. Positions of the glass pipette and wire electrodes for targeted IEP in Dp are indicated. A virtual line between the lateral edge of the telencephalon and the midline (white) was used to determine the position of the injection pipette along the medial-lateral axis (Methods). **(D)** Approximate positions of electrodes (black) and injection sites (orange) for EEP in the dorsal telencephalon (left) and targeted IEP in Dp (right). Plasmid was injected and electroporated sequentially at three different depths (gray lines). **(E)** Needle electrodes for “external-electrode-electroporation” (EEP; left) and wire electrodes for “internal-electrode-electroporation” (IEP; right). Insets show electrical pulse protocols.

For surgical procedures, fish were anesthetized with 0.01% tricaine methanesulfonate (MS-222, Sigma-Aldrich). Larvae were embedded in low-melting agarose using standard procedures. Adult fish were held dorsal side up by a fish holder made from wet sponges inside a flexible plastic tube. The body of the fish was held by the sponges while the head was free. The tube was integrated in a custom-made stereotactic chamber with lateral stabilizers that were used when high spatial precision and stability was required. The chamber was placed on a tilted stage under a stereomicroscope (Olympus SZX16 or Wild; Figure 1A left). A cannula was inserted into the mouth of the fish to continuously apply fresh fish water with MS-222 to the mouth and gills. The skin was kept wet by regular supply of fish water. After surgery, fish were returned to standard tanks.

In order to monitor expression of fluorescent proteins through the skull, fish were anesthetized with MS-222 and mounted as described above. Fish were then imaged from the dorsal side using an Olympus SZX16 fluorescence stereomicroscope equipped with a color CCD camera (Olympus) and returned to their home tanks afterwards.

### HSV-1 and DNA constructs

HSV-1vectors were obtained from three different sources: (1) BioVex (USA; kindly provided by Dr. J. Letzkus), (2) SinoGenomax (China), (3) the Massachusetts Institute of Technology (MIT) viral core (USA). Note that sources (1) and (2) have recently discontinued the custom production of HSV-1. All HSV-1 viruses used in this study and their inserts, sources, titers, and production methods (Simonato et al., 1999) are summarized in Table 1.

**Table 1:** **HSV-1 viruses and expression in the dorsal telencephalon of adult zebrafish**.

**No**.	**Virus insert (promoter :: gene)**	**Virus source**	**Titer (units/ml)**	Production method^*^	**Number of fish**	**Expression strength**
1	hEF1α::GFP	BioVex	n.a.	Amplicons	*n* = 9	+++
2	hEF1α::ChR2-2A-NpHR2.0YFP	BioVex	1.4 × 10^10^	Amplicons	*n* = 3	−
3	CMV::GFP	SinoGenomax	2 × 10^8^	Replication-defective vector	*n* = 4	+
4	hEF1α::GFP	SinoGenomax	2 × 10^8^	Replication-defective vector	*n* = 4	−
5	ST-IE4/5::DsRed2	MIT viral core	3 × 10^8^	Amplicons	*n* = 8	++
6	ST-CMV::GFP	MIT viral core	3 × 10^8^	Amplicons	*n* = 4	+
7	LT-CMV::DsRed2	MIT viral core	3 × 10^8^	Amplicons	*n* = 10	+++
8	CaMKII::GFP	MIT viral core	3 × 10^8^	Amplicons	*n* = 4	−
9	rEF1α::GFP	MIT viral core	3 × 10^8^	Amplicons	*n* = 4	−
10	hEF1α::GFP	MIT viral core	3 × 10^8^	Amplicons	*n* = 4	+
11	LT-CMV::RG-GFP	MIT viral core	4.5 × 10^8^	Amplicons	*n* = 4	+++

hEF1α, human elongation factor 1 alpha; CMV, cytomegalovirus immediate-early gene; ST- IE4/5, immediate early gene 4/5 promoter with short-term expression; ST-CMV, CMV promoter with short-term expression; LT-CMV, CMV promoter modified for long-term expression; CaMKII, Ca^2+^/calmodulin-dependent protein kinase II; rEF1α, rat elongation factor 1 alpha; 2A, self-processing viral peptide cleavage site for co-expression of multiple polypeptides; RG-GFP, fusion of rabies virus glycoprotein and GFP; n.a., not available. For further information on viruses from MIT Viral Core see mcgovern.mit.edu/technology/viral-core-facility.

* For further information on production methods see Simonato et al. (1999). Titers of HSV-1 from MIT Viral Core have been estimated based on previous measurements but not measured directly for each batch. Expression strength was scored on a scale ranging from no detectable expression (−) to strong expression (+++).

Plasmids used for electroporation are summarized in Table 2. Self-made constructs were generated from the components described by standard procedures including PCR, restriction cloning, and the gateway system (Kwan et al., 2007). For *in vivo* electroporation, plasmids were dissolved in calcium-free Ringer's solution (NaCl 119 mM, KCl 2.9 mM, HEPES 5 mM; pH 7.2) or, in a few cases, in 0.9% NaCl. Plasmid concentrations were between 0.2 μg/μl and 4 μg/μl. In most experiments, a concentration of approximately 1 μg/μl was used. Co-electroportation of two plasmids was performed using equal concentrations of each plasmid.

**Table 2:** **Plasmids used for electroporation**.

**No**.	**Plasmid (promoter :: gene)**	**Description/source/references**
1	hEF1α::GFP	The plasmid was constructed by combining the human EF1α promoter (Kim et al., 1990) (Gift from C. Xu) with green fluorescent protein (GFP).
2	hEF1α::ChR2tc-GFP	The plasmid was constructed based on plasmid #1. ChR2tc is a ChR2 mutant with the T159C mutation, which increases the photocurrent (Berndt et al., 2011). ChR2tc cDNA was a gift from T. Oertner and fused to GFP.
3	hEF1α::ChR2tc-mEos2	The plasmid was constructed based on plasmid #1 and ChR2tc-mEos2, a gift from T. Oertner. ChR2tc-mEos2 is a fusion of ChR2tc (described above) and the photoconvertible fluorescent protein mEos2 (McKinney et al., 2009), Addgene 20341.
4	xEF1α::GFP	The plasmid was constructed by combining the Xenopus EF1α promoter (Johnson and Krieg, 1994) (gift of K. Kawakami) with GFP.
5	zHsp70l::GFP	The plasmid was constructed by combining the zebrafish Hsp70l promoter (Halloran et al., 2000) from the Tol2-kit (Kwan et al., 2007) with GFP.
6	zHsp70l::GCaMP5	The plasmid was constructed by combining the zebrafish zHsp70l (Halloran et al., 2000) from the Tol2-kit (Kwan et al., 2007) with GCaMP5, a green fluorescent calcium indicator (Akerboom et al., 2012). GCaMP5 cDNA was a gift from L. Looger and D. Kim.
7	CAG::Cre-GFP	CAG is a chimeric promoter (Miyazaki et al., 1989), Cre-GFP is a recombinase fused to GFP (Matsuda and Cepko, 2007).
8	αCaMKII::GFP(1)	Gift from A. Fine (Mayford et al., 1996). The plasmid contains a short version of the αCaMKII promoter (0.4 kb) and GFP.
9	αCaMKII::GFP(2)	Gift from A. Fine (Mayford et al., 1996). The plasmid contains a longer version of the αCaMKII promoter (1.3 kb) and GFP.
10	hSyn::ChR2wt-GFP-mbd	Gift from S. Wiegert and T. Oertner; the plasmid contains the human Synapsin-1 promoter (Kügler et al., 2003) and wild type ChR2 fused to GFP and a myosin binding domain (mbd) that can target ChR2 to the somato-dendritic compartments (Lewis et al., 2009).
11	zElavl3::GCaMP5	Gift from A. Schier. The plasmid contains the zebrafish Elavl3 (HuC) promoter (Park et al., 2000) and the GECI GCaMP5 (Akerboom et al., 2012).
12	zElavl3::itTA	The plasmid contains the zElavl3 promoter and the Tet activator (itTA), a transcription activator that binds specifically to tet operator (tetO) (Zhu et al., 2009).
13	tetO7::ChR2wt-YFP	The plasmid contains seven repeats of the tet operator with a minimum CMV promoter (tetO7) and wild type ChR2 fused to yellow fluorescent protein (YFP) (Zhu et al., 2009).
14	CMV::mRuby	The plasmid contains the CMV promoter (Thomsen et al., 1984) and mRuby, a monomeric red fluorescent protein (Kredel et al., 2009).
15	CMV:: mGFP-αCaMKII	The alpha Ca^2+^ /calmodulin-dependent protein kinase II (αCaMKII) gene was fused to monomeric GFP (Hudmon et al., 2005).
16	CMV::GCaMP6f	Obtained from Addgene 40755 (Chen et al., 2013). GCaMP6f is a green fluorescent calcium indicator with fast kinetics.
17	CMV::GCaMP6s	Obtained from Addgene 40753 (Chen et al., 2013). GCaMP6s is a green fluorescent calcium indicator with slow kinetics.
18	CMV::RGECO1.0	The plasmid contains the CMV promoter and RGECO1.0, a red fluorescent GECI (Zhao et al., 2011).
19	CMV::RCaMP1.07	The plasmid contains the CMV promoter and RCaMP1.07, a red-fluorescent GECI (Ohkura et al., 2012).

hEF1α, human elongation factor 1 alpha; xEF1α, Xenopus elongation factor 1 alpha; zHsp70l, zebrafish heat-shock protein 70l; CAG, chimeric promoter with sequences from cytomegalovirus immediate-early gene, chicken beta-actin gene, and rabbit beta-globin gene; αCaMKII, Ca^2+^/calmodulin-dependent protein kinase II; hSyn, human synapsin-1 gene; zElavl3, zebrafish Elavl3 (HuC) gene; CMV, cytomegalovirus immediate-early gene; tetO7, minimum CMV promoter with seven repeats of tet operator. Other abbreviations are explained in the right column.

### Stereotactic procedures in adult fish and microinjection of viral vectors

Virus suspensions were injected into the dorsal telencephalon (areas Dm, Dc, and/or Dl), the olfactory bulb, or Dp. All procedures were performed under a stereomicroscope. Experiments in the dorsal telencephalon did not require high spatial precision. In these cases, the fish was held by the sponge holder without lateral stabilizers. A craniotomy was made over the dorsal telencephalon near the midline using a dentist's drill. Micropipettes were inserted vertically through the craniotomy into the dorsal telencephalon using a manual 3-axis manipulator (WPI; Figure 1A). Care was taken to avoid major blood vessels. Three injections of 50 to a few 100 nl were made 250, 350, and 450 μm below the level of the bone.

Injections into the olfactory bulb or Dp were performed using the stereotactic chamber and lateral stabilizers. Dp was targeted by a stereotactic procedure that was developed based on the zebrafish brain atlas (Wullimann and Reichert, 1996). Hematoxylin and eosin (H&E) staining of coronal, horizontal and sagittal brain sections through Dp were performed to confirm the cell body distribution within Dp and the position of Dp relative to the skull (Figure 1B). A craniotomy was made on the suture between the bones over the telencephalon and tectum. In the lateral-medial direction the craniotomy was located approximately 25% along a virtual line between the lateral edge of the telencephalon and the midline (Figure 1C). A micropipette containing virus suspension was inserted through the craniotomy slightly anterior to the suture, avoiding blood vessels (Figure 1C, orange dot). Three injections were made approximately 400, 500, and 600 μm below the level of the bone (Figure 1D). The precise depths of injection points were adjusted slightly based on the size of each fish. In order to target injections to the olfactory bulb a craniotomy was made at the anterior edge of the telencephalic skull (Figure 1C) and virus was injected 200, 300, and 400 μm below the level of the bone.

Virus suspensions were injected using glass micropipettes with a long shaft that were prepared from borosilicate capillaries (1 mm diameter, Hilgenberg) using an electrode puller (P-2000, Sutter). The tip was broken to obtain a diameter of 10–20 μm. At each injection point, the capillary was pressurized using a syringe connected with flexible tubing and the ejected volume was measured by monitoring the movement of the meniscus inside the capillary.

### Electroporation

Stereotactic procedures for electroporation were equivalent to those used for viral injections. For electroporation in the dorsal telencephalon using external electrodes, 100–300 nl of plasmid suspension was injected at each of three injection points approximately 250, 350, and 450 μm below the level of the bone (Figure 1D, left). The glass pipette was then retracted and a pair of parallel sharp steel electrodes (Figure 1E left; 0.5 mm diameter), separated by approximately 1 mm, was positioned so that one electrode was placed on the craniotomy and the other was located between the eye and the skull. Electrodes were custom made from steel needles (BTX, USA) and not insulated. Electrical pulses (5 × 25 ms, 70 V, 1 Hz, square; Table 3 and Figure 1E, left) were applied with a NEPA21 electroporator (NEPAGENE, Japan) or a Gene Pulser Xcell electroporator (Bio-Rad, USA). The delay between DNA injection and electrical stimulation was approximately 20 s. This procedure is relatively simple, reliable, and allows for the detection of fluorescent protein expression through the intact skull using a fluorescence stereomicroscope. The procedure was used to analyze the time course of protein expression *in vivo* and to test the efficiency of different promoters.

**Table 3:** **Pulse settings for electroporation**.

**No**.	**Tissue impedance**	**Poring pulse**					**Transferring pulse**				
		**Voltage (V)**	**Pulse duration (ms)**	**Interval (ms)**	**Number of pulse**	**Polarity switch**	**Voltage (V)**	**Pulse duration (ms)**	**Interval (ms)**	**Number of pulse**	**Polarity switch**
**GENE PULSER XCELL ELECTROPORATOR, FOR EEP**											
1	n.a.	n.a.	n.a.	n.a.	n.a.	n.a.	70	25	1 s	5	No
**NEPA21 ELECTROPORATOR, FOR EEP**											
2	n.a.	100	0.1	999.9	2 × 1	Yes	20	5	95	2 × 25	Yes
**NEPA21 ELECTROPORATOR, FOR IEP, PORING VOLTAGE CALCULATED FOR MAXIMUM CURRENT OF 6 mA**											
3	6–9 kΩ	36	0.1	999.9	2 × 1	Yes	7.2	1	99	2 × 50	Yes
4	9–12 kΩ	54	0.1	999.9	2 × 1	Yes	10.8	1	99	2 × 50	Yes
5	12–16 kΩ	72	0.1	999.9	2 × 1	Yes	14.4	1	99	2 × 50	Yes
6	16–20 kΩ	96	0.1	999.9	2 × 1	Yes	19.2	1	99	2 × 50	Yes
7	20–25 kΩ	120	0.1	999.9	2 × 1	Yes	24	1	99	2 × 50	Yes
8	25–30 kΩ	150	0.1	999.9	2 × 1	Yes	30	1	99	2 × 50	Yes
9	30–36 kΩ	180	0.1	999.9	2 × 1	Yes	36	1	99	2 × 50	Yes
10	36–42 kΩ	216	0.1	999.9	2 × 1	Yes	43.2	1	99	2 × 50	Yes
11	42–50 kΩ	252	0.1	999.9	2 × 1	Yes	50.4	1	99	2 × 50	Yes
12	>50 kΩ	300	0.1	999.9	2 × 1	Yes	60	1	99	2 × 50	Yes

n.a., not applicable. Settings #6 and #7 were used most frequently for IEP.

Targeted electroporation in Dp using internal electrodes was performed using lateral stabilizers in the stereotactic chamber. DNA solution was loaded into a micropipette that was held vertically by a manual 3-axis manipulator as described above (Figure 1A). A pair of custom-made parallel thin Pt electrodes (25 μm diameter, approximately 400 μm distance, shank insulated, tip exposed, modified from FHC Inc. CE2C40; Figure 1E, right) was mounted on a second 3-axis manual manipulator. Electrodes were almost parallel to the micropipette (Figure 1A right) and positioned so that the injection pipette was between the electrodes above the craniotomy. The glass pipette and the pair of electrodes were then inserted together into the tissue. Three injections were made approximately 400, 500, and 600 μm below the level of the bone (Figure 1D). At each injection point, approximately 70 nl of DNA solution was ejected and the tissue impedance was measured immediately afterwards. Based on the measured impedance, a set of pre-programmed square electrical pulses was selected (Table 3) and applied 1–2 times immediately after DNA injection using the NEPA21 electroporator.

The two electroporators used in this study included a basic instrument (Gene Pulser Xcell, Bio-Rad, USA) and a more advanced instrument (NEPA21, NEPAGENE, Japan). Targeted local electroporation was performed exclusively using the NEPA21 electroporator because this instrument allowed for fine tuning of the pulse protocol based on tissue impedance. Pulse trains consisted of a pair of high-amplitude poring pulses with opposite polarity followed by a train of lower-amplitude transfer pulses. The polarity of the transfer pulses was reversed after 50% of pulses were applied (Figure 1E, right). The amplitude of the pulses was adjusted based on tissue impedance, which was measured using the NEPA21 electroporator. Highest cell survival and expression levels were obtained when the voltage of the poring pulse was set to yield currents of 4–6 mA and the voltage of the transfer pulses was 20% of that of the poring pulse. The pulse duration was kept short (0.1–1 ms) in order to avoid accumulation of heat. For tissue with an impedance of 16–20 kΩ, for example, the pulse train consisted of a pair of square pulses of 0.1 ms and ±96 V for membrane poring followed by 50 square pulses of 1 ms, 19.2 V and 10 Hz for DNA transfer and another 50 square pulses with the same parameters but opposite polarity (Table 3). In order to minimize the time delay between impedance measurements and pulse application, predefined pulse trains were stored in the memory of the electroporator (Table 3).

### *Ex-vivo* preparation, multiphoton imaging, electrophysiology, odor application, and optical stimulation

Multiphoton imaging and electrophysiological experiments were performed in an *ex-vivo* preparation of the adult zebrafish brain as described (Zhu et al., 2012). Briefly, fish were cold-anesthetized, decapitated, and the dorsal or ventral forebrain was exposed. The preparation was then transferred to a custom-made imaging chamber, continuously perfused with teleost artificial cerebrospinal fluid (ACSF) (Mathieson and Maler, 1988), and warmed up to room temperature.

High-resolution imaging of fluorescent protein expression and calcium signals were performed using a custom-made multiphoton microscope that was constructed around the body of an Olympus BX-51 microscope. The microscope was equipped with a 20× water immersion objective (NA 0.95, Olympus), a Ti:Sapphire laser (Spectra Physics, USA), a custom-built unit containing galvanometric scanners (6215H, Cambridge Technology, USA) and custom-built external detection optics with photomultipliers (H7422P-40MOD, Hamamatsu). GFP/YFP were excited at 860 or 980 nm; red-fluorescent proteins were excited at 980 nm. Fluorescence emission was detected in two channels using green (535/50 nm) and red (640/75 nm) emission filters. A third channel was used to acquire the signal of a position-sensitive detector for transmitted infrared light. This channel produced a contrast-enhanced transmitted light image that was used to direct the recording pipette for patch clamp recordings. The microscope and related equipment were controlled by ScanImage and Ephus software (Pologruto et al., 2003; Suter et al., 2010). For calcium imaging, series of fluorescence images were collected at 128 ms/frame, in some cases 512 ms/frame or 64 ms/frame, for approximately 20 s. Laser intensity was adjusted to minimize photobleaching.

Whole-cell patch clamp recordings were performed using borosilicate pipettes (8–12 MΩ), a Multiclamp 700 B amplifier (Molecular Devices) and Ephus software (Suter et al., 2010). Neurons were targeted by a combination of multiphoton fluorescence and contrast-enhanced transmitted-light optics (transmitted light channel). Pipettes were filled with an intracellular solution containing 130 mM potassium gluconate, 10 mM sodium gluconate, 10 mM sodium phosphocreatine, 4 mM sodium chloride, 4 mM magnesium-ATP, 0.3 mM sodium-GTP, 10 mM HEPES (pH 7.2, 300 mOsm) and 10 μ M Alexa Fluor 594 (Invitrogen). Signals were digitized at 10 kHz.

Electrical stimulation in the olfactory bulb was performed by placing a glass pipette (tip diameter, 30–50 μm) filled with 1 M NaCl at the posterior end of the olfactory bulb. A train of 10 pulses (0.5 ms pulse width, −35 V, 20 Hz) was delivered 10 times at an inter-trial interval of 12 s. In order to induce slow, epileptiform population activity, the GABA_A_ receptor antagonist Gabazine (1 μM) was added to the ACSF.

Optical stimulation of ChR2 with blue light was performed with a strong LED (460 nm; Luxeon, USA) that was mounted in the epifluorescence lamphouse attached to the Olympus BX-51 microscope body. Optical stimuli consisted of trains of light pulses (10 ms duration; 10 pulses at 5 or 10 Hz; light power under objective approximately 250–300 μW/mm^2^). At least 15 trials were acquired for each cell. In some electrophysiological recordings, optical stimulation caused a small stimulus artifact that was removed from the recorded traces by replacing voltage values with a constant value.

Odors were delivered through a constant stream of carrier medium directed at the ipsilateral naris using a computer controlled HPLC injection valve (Rheodyne, USA) as described (Tabor et al., 2004). Odor stimulation was repeated at least three times with an inter-trial interval of at least 2 min to avoid adaptation. Food extract was prepared from standard dry fish food (SDS, UK) as described (Tabor et al., 2004) and diluted 1:1000 before the experiment.

### Data analysis

Electrophysiology or calcium imaging data were analyzed using custom routines written in Matlab or IGOR Pro. Synaptic currents evoked by optical stimulation were measured by whole-cell voltage clamp recordings and averaged over 150 pulses (15 trials with 10 pulses each). The synaptic latency was estimated as the time between the offset of the 10 ms light pulse and the first inflection of the current trace. The inflection point was usually sharply defined within a time window of < 2 ms and determined manually by inspection of each trace. The amplitudes of averaged EPSCs and IPSCs were measured as the peak currents within a 20 ms time window after the offset of the light pulse relative to pre-stimulus baseline.

Calcium signals (ΔF/F) were calculated as changes in fluorescence intensity (ΔF) relative to a baseline period of 2–4 s before response or stimulus onset (F). To quantify calcium signals of individual neurons, regions of interest were outlined manually on time-averaged ΔF/F maps. In order to quantify fluorescence changes of GECIs during epileptiform activity, 9 neurons from 3 fish were analyzed for each GECI. In each neuron, ΔF/F of calcium transients were measured at the soma and at a dendritic location that showed large ΔF/F values in time-averaged maps. Amplitudes of multiple large calcium transients were then averaged for each neuron, and mean calcium transients were then averaged over neurons for each GECI.

In order to assess the fluorescence intensity in the dorsal telencephalon through the intact skull, individual fish were anesthetized and viewed through a fluorescence stereomicroscope. Fluorescence intensity was scored manually relative to the mean fluorescence intensity observed 10 days after electroporation of plasmid #10. This plasmid contained the hSyn promoter and was chosen as a reference because it produced an intermediate fluorescence intensity.

## Results

### *in vivo* gene transfer using HSV-1

Replication-incompetent HSV-1 viruses have been used successfully as vectors to express transgenes in the brain of rodents and other vertebrates (Palella et al., 1989; Luo et al., 2008; Yonehara et al., 2011). Because many HSV-1 viruses infect neurons retrogradely via their axon terminals they can be used to target projection neurons by injections into their terminal areas (Ugolini et al., 1987; Yonehara et al., 2011). We tested the ability of 11 replication-incompetent HSV-1 viruses (Table 1) to express transgenes in neurons of the adult zebrafish brain. Using stereotactic procedures, HSV-1 viruses were injected through small craniotomies into the dorsal telencephalon, into Dp, or into the olfactory bulb (Methods). Each HSV-1 virus was injected into 3–10 fish. Expression was scored *in vivo* by imaging fluorescence through the intact skull at different time points after injection. In addition, high resolution images of neurons expressing fluorescent markers were obtained by multiphoton microscopy in an *ex-vivo* preparation of the brain (Zhu et al., 2012).

**Figure 2:**
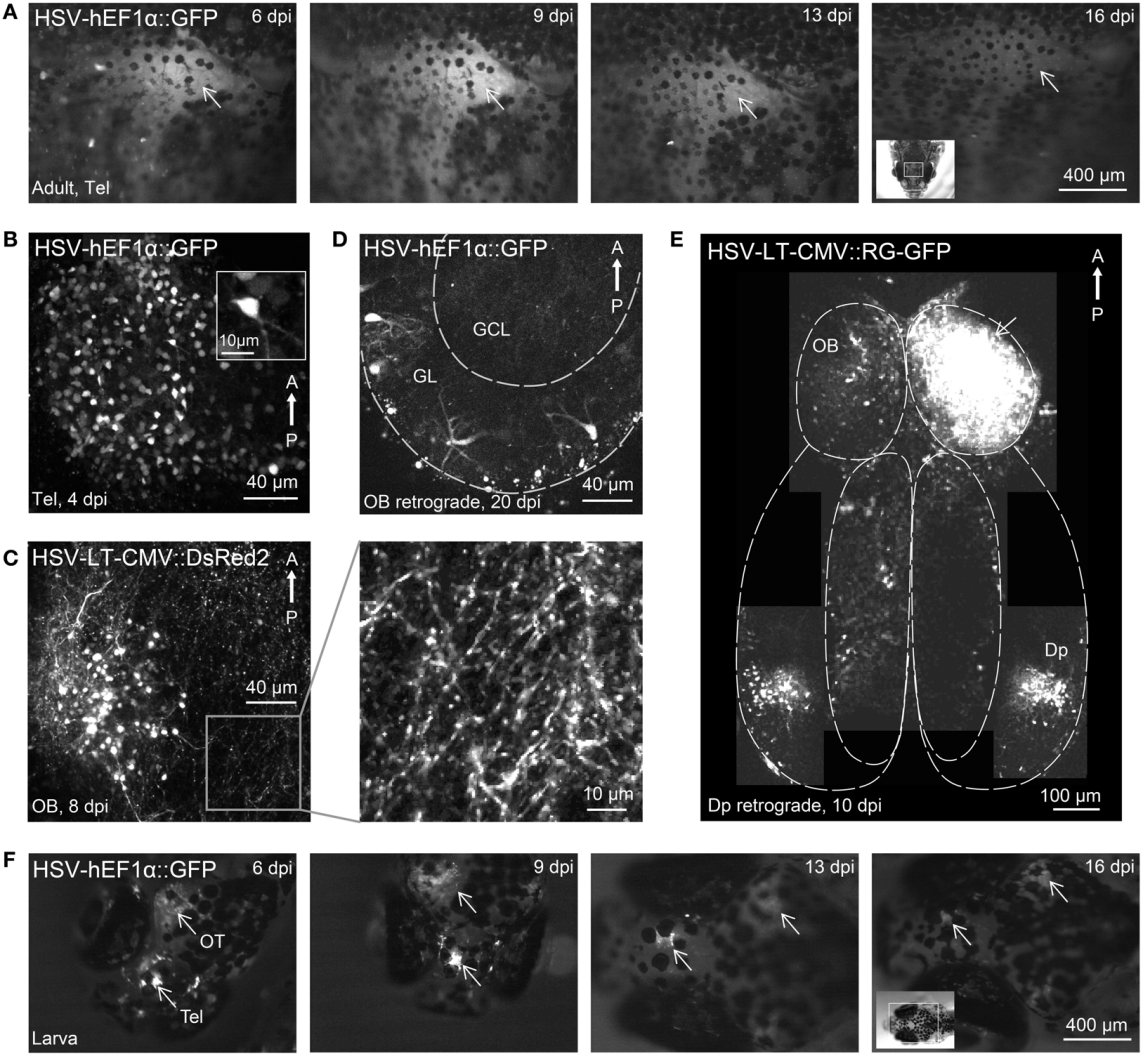
**Gene expression in the zebrafish brain using HSV-1. (A)** Fluorescence images of the dorsal head of an adult zebrafish at different time points after injection of HSV-1 (#1). Images were taken with a fluorescence stereomicroscope; arrow indicates region of strong fluorescence. **(B)** Telencephalic neurons expressing GFP 4 days after injection of HSV-1 into the dorsal telencephalon (#1; *z*-projection of multiphoton stack). **(C)** Olfactory bulb neurons expressing DsRed2 8 days after injection of HSV-1 into the olfactory bulb (#7; *z*-projection of multiphoton stack). Boxed region is shown at higher magnification on the right. **(D)** Transgene expression in olfactory bulb neurons, presumably mitral cells, 20 days after injection of HSV-1 (#1) into Dp. GL, glomerular/mitral cell layer; GCL, granule cell layer. **(E)** Composite image (multiple z-projections of multiphoton stacks) showing transgene expression throughout the ventral forebrain after injection of HSV-1 (#11) into one olfactory bulb (arrow). Note strong bilateral expression in Dp but not in other telencephalic areas. **(F)** Fluorescence images of the dorsal head of a zebrafish larva at different time points after injection of HSV-1 (#1). Virus was injected at two sites, the telencephalon (Tel) and the optic tectum (OT). Arrows indicate strong fluorescence around the injection sites.

A subset of HSV-1 viruses (e.g., #1, #7, and #11; Table 1; Figures 2A–C) produced dense and robust expression of fluorescent marker proteins while injection of other HSV-1 viruses produced weak or no detectable fluorescence (e.g., #2, #4, #8, and #9; Table 1). These differences were consistently observed in multiple experiments, indicating that they were not caused by stochastic factors such as variable success of injections. Moreover, independent batches of one of the HSV-1 viruses (#7) produced consistent results, suggesting that variation in the efficiency of virus production is unlikely to account for the observed differences. Generally, HSV-1 viruses produced by replication-defective vectors resulted in less fluorescence than HSV-1 viruses produced by amplicons, suggesting that viral infection, transgene expression or cell survival depend on the method of virus production (Simonato et al., 1999). In addition, expression may depend on promoters, transgenes, titers, and other factors.

Most HSV-1 viruses produced fluorescence that could be observed through the intact skull. Fluorescence was first observed 2 days post-injection (dpi), reached a maximum around 9 dpi and declined slowly thereafter (Figure 2A), often lasting more than 4 weeks. Expression driven by a CMV promoter for short-term expression (#6) decayed rapidly after approximately 7 dpi while expression driven by a CMV promoter designed for long-term expression (#7) remained high even 28 dpi (not shown). High resolution imaging of infected neurons revealed fluorescence in many somata and neuronal processes (Figures 2B,C). No fragmented cells, fluorescent aggregates or other obvious signs of cell death were observed, and no obvious tissue damage was apparent. Injection of 100–200 nl of virus #1 caused transgene expression in approximately 150 ± 50 cells (*n* = 4 fish, mean ± SD) within a volume of approximately 200 × 200 × 100 μm^3^ around the injection site.

Injections of HSV-1 virus into Dp labeled neurons in the outer layer of the olfactory bulb where mitral cells projecting to Dp are located (Figure 2D, *n* = 3 fish). Few labeled neurons were found in telencephalic areas between the olfactory bulb and Dp and no labeled neurons were found in deep layers of the olfactory bulb (Figure 2D), which contain large numbers of local granule cells. Injections into the olfactory bulb labeled somata in Dp, which provides strong bilateral projections to the olfactory bulb (Figure 2E). Very few labeled cells were seen in telencephalic areas between the olfactory bulb and Dp. Hence, HSV-1 vectors can infect projection neurons retrogradely via their axons, consistent with observations in other species.

Injection of HSV-1 into the larval brain (virus #1; *n* = 10 fish) also produced robust fluorescence for > 2 weeks around the injection site (Figure 2F). These results show that modified HSV-1 viruses are efficient tools for gene transfer into the zebrafish brain.

### Gene transfer by electroporation

Electroporation has been used in zebrafish to introduce DNA constructs into larval neurons, adult retinal neurons or adult muscle cells (Rambabu et al., 2005; Hendricks and Jesuthasan, 2007; Bianco et al., 2008; Kustermann et al., 2008). However, the procedures used in these studies cannot be used to express transgenes in the adult zebrafish brain without major modifications.

We first developed a simple procedure to electroporate plasmids into neurons of the dorsal telencephalon without high spatial precision. Plasmid DNA (hEF1α::GFP, plasmid #1, Table 2; 200–500 nl) was injected into the dorsal telencephalon through a small craniotomy. After withdrawal of the injection pipette, a pair of electrodes was placed outside the skull, flanking the injection site, and train of voltage pulses was delivered (Methods; Figure 1D, left). Fish were then returned to their home tanks and inspected for fluorescence through the intact skull at successive time points. Fluorescence was observed after a few days even in pilot experiments before optimization of experimental parameters. Based on these initial observations we tested different electrodes, electrode positions, pulse settings, DNA concentrations and solvents to maximize the observable fluorescence signal. Strong expression was achieved with a pair of parallel, sharp stainless steel needles, separated by approximately 1 mm (Figure 1E, left; Methods), when one electrode was located near the craniotomy and the other was located near the edge of the ipsilateral eye (Figure 1D, left). The preferred pulse protocol consisted of five square pulses of 25 ms delivered at 1 Hz with an amplitude of 70 V (Figure 1E, left). DNA was usually dissolved in Ca^2+^-free Ringer solution at a concentration of 1 μg/μl (Methods). We refer to this approach as “external-electrode-electroporation” (EEP).

**Figure 3:**
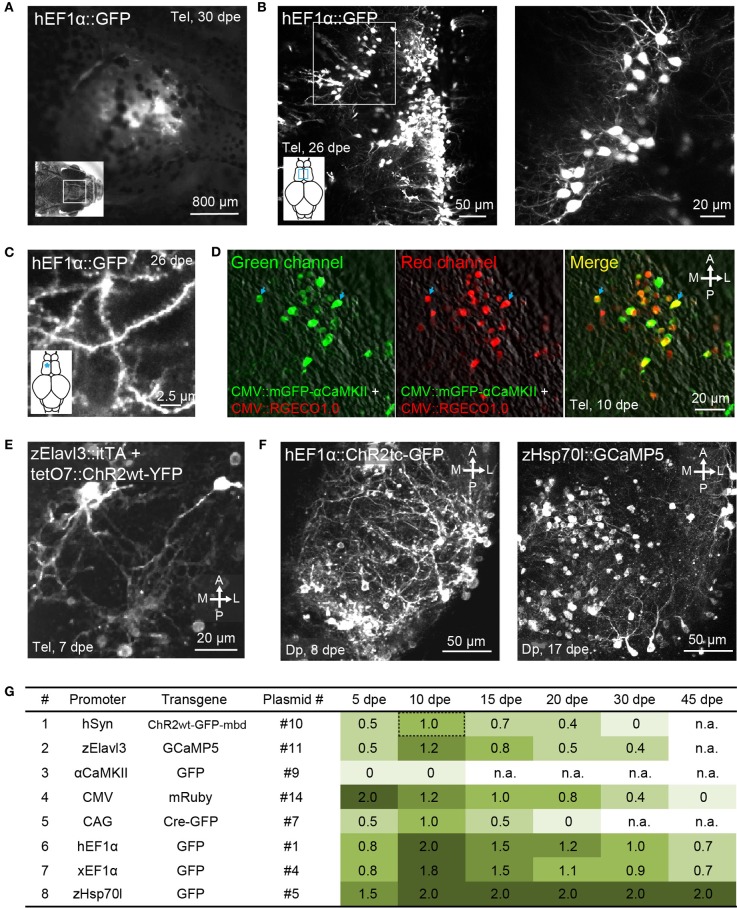
**Gene expression in the adult zebrafish brain using electroporation. (A)** Fluorescence image of the dorsal head of an adult zebrafish 30 days after electroporation (dpe) of plasmid #1 (EEP; hEF1α::GFP). Image was taken with a fluorescence stereomicroscope. **(B)** Expression of GFP in the dorsal telencephalon after electroporation of plasmid #1 (EEP; *z*-projection of multiphoton image stack). Boxed area is shown at higher magnification on the right. **(C)** GFP expression in spiny dendrites (same fish as in **B**; location is indicated by asterisk). **(D)** Expression of mGFP-αCaMKII (green channel, left) and RGECO1.0 (red channel, center) after co-electroporation of plasmids #15 and #18 (EEP in the dorsal telencephalon). Right: overlay showing co-expression. **(E)** Expression of ChR2wt-YFP after co-electroporation of a plasmid harboring the Tet activator (itTA; #12) and another plasmid containing the responder element (tetO7::ChR2wt-YFP; #13; EEP in the dorsal telencephalon). **(F)** Expression of ChR2tc-GFP (plasmid #2; left) and GCaMP5 (plasmid #6; right) in Dp after targeted electroporation using internal wire electrodes (IEP; z-projections of multiphoton image stacks). **(G)** Fluorescence intensity observed through the dorsal skull at different time points after electroporation of different constructs (EEP in dorsal telencephalon). Fluorescence intensity was scored manually through a fluorescence stereomicroscope and normalized to the intensity observed 10 days after electroporation of plasmid #10, which contains promoter #1 (hSyn::ChR2wt-GFP-mbd; Methods). n.a, not analyzed.

Using EEP in the dorsal telencephalon and plasmid #1 (hEF1α::GFP), strong and widespread fluorescence was observed through the intact skull in 11/12 fish. Fluorescence was first detected 3 days post-electroporation (dpe), peaked around 8–12 dpe, and thereafter remained high for weeks (Figures 3A,B). Multiphoton imaging in the dorsal telencephalon showed strong GFP expression in hundreds of cells (estimated number: 650 ± 250 cells/fish, mean ± *SD*; *n* = 4 fish; Figure 3B). GFP-expressing neurons were distributed throughout a large volume, sometimes the entire dorsal side of the telencephalic hemisphere. Neurons had normal morphologies without obvious signs and no obvious signs of cell death or tissue damage were observed. Some neurons had long processes, consistent with previous anatomical descriptions of neurons in the dorsal telencephalon (Aoki et al., 2013), and some neurons had spiny dendrites (Figure 3C).

Co-electroporation of two plasmids harboring reporters of different colors frequently resulted in overlapping expression of the reporters in the same cells (plasmids #15 and #18; Figure 3D; *n* = 3 fish). Moreover, co-electroporation of a plasmid containing a Tet driver construct (plasmid #12) and a second plasmid containing a Tet responder construct (plasmid #13) resulted in transgene expression in a substantial number of cells (Figure 3E). Hence, electroporation can be used to co-express multiple transgenes from different plasmids, consistent with previous observations (Barnabé-Heider et al., 2008).

When plasmids were injected into Dp, the same electroporation protocol produced no detectable expression within Dp although some labeled neurons were found near the craniotomy in the dorsal telencephalon. EEP is therefore not equally effective throughout the brain, implying that gene transfer by EEP cannot easily be targeted and confined to small brain areas. Dp is located approximately 400–600 μm below the dorsal skull (Figure 1B, bottom) next to a prominent bone, suggesting that the efficiency of electroporation is non-uniform because the electrical field is distorted by inhomogeneities of the tissue, particularly around bones. In order to overcome these problems we fabricated pairs of electrodes from insulated Pt wires with a diameter of 25 μm. Wires were glued together in parallel with a spacing of approximately 400 μm and the insulation was removed only at the tips. Electrodes were then inserted into the brain together with a glass pipette containing the plasmid suspension. The wire electrodes and the pipette were targeted to Dp using a stereotactic procedure (Methods), plasmid suspension was injected between the two wire electrodes, and voltage pulses were applied across the electrodes. This injection and electroporation sequence was repeated at three different depths in Dp, spaced by approximately 100 μm (Methods; Figure 1D, right).

Targeted electroporation using internal electrodes resulted in expression of fluorescent markers in Dp. In most cases, the expression was completely restricted to a volume of approximately 200 × 200 × 200 μm^3^ within Dp. The careful design of voltage pulse trains can reduce damage and substantially enhance the efficiency of electroporation (Šatkauskas et al., 2012). Generally, it is recommended to use pulse trains consisting of a pair of brief, high-amplitude poring pulses of opposite polarity to permeabilize the plasma membrane followed by a train of longer pulses with lower amplitude and changing polarity to transfer the DNA into the cell. Furthermore, it is useful to adjust the amplitude of voltage pulses to the tissue impedance in each experiment in order to generate a reproducible current (Šatkauskas et al., 2012). We found that these procedures considerably improved electroporation results as compared to simpler pulse trains. Best results were obtained when the calculated poring currents were 4–6 mA and when the pulse trains were designed as specified in Table 3. This optimized protocol resulted in reliable expression of transgenes that lasted for weeks (see below). We refer to this protocol as “internal-electrode-electroporation” (IEP).

Using IEP and plasmid #1 (hEF1α::GFP; *n* = 7 fish) or plasmid #2 (hEF1α::ChR2tc-GFP; *n* = 40 fish), reporter gene expression in Dp was observed in 85% of fish (Figure 3F, left). In a subset of fish electroporated with plasmid #2, fluorescent neurons were counted throughout Dp. On average, reporter expression was detected in 23 ± 5 cells per Dp (mean ± SD; *n* = 26 fish). The morphology of GFP-expressing neurons was normal without obvious signs of damage. In most cells, expression appeared strong compared to the expression of the same or similar transgenes in stable transgenic lines (not shown). Strong fluorescence was observed even for transgenes that are usually difficult to express at high levels such as fusion proteins containing ChR2 (Figure 3F, left). IEP is therefore a fast and reliable method to express transgenes in spatially restricted populations of neurons at high levels.

### Characterization of promoters for expression in the adult zebrafish brain

The intensity, cell type specificity and time course of transgene expression is expected to depend critically on the promoter in an expression construct. In stable transgenic lines, many promoters drive much broader expression at early developmental stages than in the adult brain (Stamatoyannopoulos et al., 1993; Goldman et al., 2001; Li et al., 2005; Zhu et al., 2009), raising the possibility that expression of transgenes in the adult brain is difficult to achieve. However, little is known about the activity of promoters when developmental processes are bypassed by introducing expression constructs directly into the adult brain. We therefore analyzed transgene expression under the control of eight promoters that drive broad expression at early developmental stages (Table 4; Figure 3G). Electroporation was preferred over HSV-1 for gene delivery because available plasmids could be used without the need to generate viral vectors.

**Table 4:** **Promoters compared by electroporation**.

**No**.	**Promoter**	**Size**	**Transgene**	**Number of fish (EEP)**	**Description**
**PAN NEURONAL EXPRESSION**					
1	hSyn	0.6 kb	ChR2wt-GFP-mbd	*n* = 5	Human synapsin-1 promoter (see plasmid #10)
2	zElavl3	8.7 kb	GCaMP5	*n* = 4	Zebrafish Elavl3 (or HuC) promoter (see plasmid #11)
**EXCITATORY GLUTAMATERGIC NEURON EXPRESSION**					
3	αCaMKII	1.3 kb	GFP	*n* = 6	Alpha Ca^2+^ /calmodulin-dependent protein kinase II promoter (see plasmid #9)
**UBIQUITOUS EXPRESSION**					
4	CMV	0.6 kb	mRuby	*n* = 5	Cytomegalovirus immediate-early promoter (see plasmid #14)
5	CAG	1.7 kb	Cre-GFP	*n* = 4	Chimeric promoter with sequences from cytomegalovirus immediate-early gene, chicken beta-actin gene, and rabbit beta-globin gene (see plasmid #7)
6	hEF1α	1.2 kb	GFP	*n* = 6	Human elongation factor 1 alpha promoter (see plasmid #1)
7	xEF1α	1.2 kb	GFP	*n* = 5	Xenopus elongation factor 1 alpha promoter (see plasmid #4)
8	zHsp70l	1.5 kb	GFP	*n* = 5	Zebrafish heat-shock protein 70l promoter (see plasmid #5)

N indicates number of fish used in EEP experiments. See Figure 3G for expression levels.

EEP was performed in the dorsal telencephalon and fluorescence was examined through the intact skull at different time points (Table 4; Figure 3G; *n* = 4–6 fish; 1 μg/μl for all plasmids). Fluorescence intensity was scored relative to the signal observed 10 days after electroporation of construct #10 (hSyn::ChR2wt-GFP-mbd), which produced intermediate expression levels. The intensity and time course of expression varied between constructs but only one promoter (αCaMKII::GFP; plasmids #8 and #9) failed to produce detectable expression. The fastest onset of expression was produced by construct #14 (CMV::mRuby), reaching peak levels at 5 dpe. Thereafter, expression gradually declined until it became undetectable at 45 dpe. Expression driven by other constructs usually peaked at 10 dpe and declined more slowly. Three constructs (#1, #4, #5) still generated substantial expression at 45 dpe. These constructs contained the human EF1α promoter (hEF1α), the EF1α promoter from Xenopus (xEF1α), and a heat-shock promoter from zebrafish (zHsp70l).

The same plasmids, along with the plasmid containing the CMV promoter (#14), also produced the highest peak fluorescence signals. Somewhat weaker but still substantial fluorescence was observed after electroporation of plasmids #7, #10, and #11, which harbored the human synapsin-1 promoter (hSyn), the zebrafish Elavl3 promoter (HuC) and the chimeric CAG promoter (Miyazaki et al., 1989), respectively. The constructs containing hSyn and zElavl3 had ChR2YFP and GCaMP5, respectively, as fluorescent reporters, which are usually less bright than the reporters of plasmids #1, #4, #5, and #14 (GFP or mRuby). The somewhat lower fluorescence generated by plasmids #10 and #11 may thus be due to the reporter, rather than the promoter. Plasmid #7 may have produced lower fluorescence because the CAG promoter is weaker than other promoters in zebrafish (Rothenaigner et al., 2011), because the reporter (Cre-GFP) is less bright, or both. Together, these results show that a broad range of promoters can drive strong and long-lasting expression of transgenes when they are introduced into the adult zebrafish brain.

The fluorescence signal produced by plasmid #5 (zHsp70l::GFP) was particularly strong and outlasted the fluorescence signals of other plasmids containing the same reporter (Figure 3G). To further examine gene expression using zHsp70l promoter, we electroporated plasmid #6 (zHsp70l::GCaMP5) into Dp by targeted IEP and found that the number of GCaMP5-expressing neurons was four times higher (92 ± 39 cells, mean ± *SD*.; *n* = 6 fish; Figure 3F, right) than the number of GFP-expressing neurons observed after electroporation of plasmid #2 (hEF1α::ChR2tc-GFP; 23 ± 5 cells, mean ± SD.; *n* = 26 fish; see above). Strong and widespread expression using the zHsp70l promoter was observed without application of a heat shock. Expression may therefore be driven by basal activity of the promoter or activated by a cellular response to the electroporation event. These results indicate that the zHsp70l promoter is particularly effective in driving expression of transgenes in a wide range of neurons when introduced into the adult zebrafish brain, consistent with previous reports (Hans et al., 2011).

### Functionality of channelrhodopsin variants and genetically encoded calcium indicators

**Figure 4:**
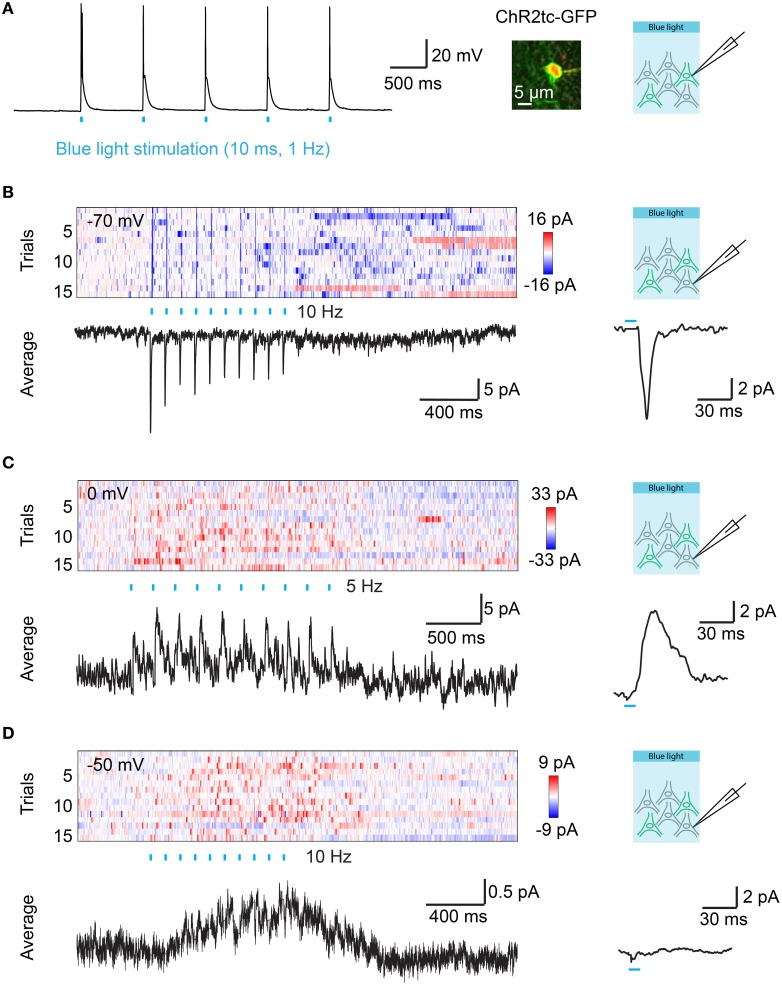
**Optical control of synaptic transmission after targeted electroporation of channelrhodopsin-2 in Dp. (A)** Action potentials evoked by blue light pulses (10 ms; 1 Hz) in a Dp neuron expressing ChR2tc-GFP after targeted IEP of plasmid #2. Image shows overlay of GFP fluorescence (green), Alexa594 fluorescence (red; included in pipette solution), and transmitted light (gray) images. **(B)** Color plot shows currents recorded at a holding potential of −70 mV in a ChR2tc-GFP-negative Dp neuron as a function of time. ChR2tc-GFP was expressed in other Dp neurons by targeted IEP of plasmid #2. Rows represent successive trials. Blue ticks indicate pulses of blue light to stimulate ChR2tc-GFP-expressing Dp neurons (10 pulses of 10 ms at 10 Hz). Black traces show the average over all 15 trials (left) and the average over all 150 individual pulses (right). The pulse-triggered current average shows a fast onset and decay, indicating that the EPSC contains a monosynaptic component. **(C)** Currents recorded at a holding potential of 0 mV in another ChR2tc-GFP-negative Dp neuron after targeted IEP of plasmid #2 (different fish). The pulse-triggered current average shows a fast onset, consistent with a monosynaptic component in the IPSC. **(D)** Currents recorded at a holding potential of −50 mV in another ChR2tc-GFP-negative Dp neuron after targeted IEP of plasmid #2 (different fish). Note a slow current but no fast EPSCs or IPSCs.

Gene transfer by HSV-1 or electroporation provides the opportunity to rapidly characterize the function of optogenetic probes, GECIs and other molecular tools in adult zebrafish. We used EEP to express four variants of ChR2, fused to fluorescent reporters, in the dorsal telencephalon of adult zebrafish (Table 2, plasmids #2, #3, #10, and #13; *n* = 4 for each plasmid). Moreover, we used targeted IEP to express plasmid #2 in Dp neurons. All constructs produced high-level expression. Labeled neurons had normal morphologies except for those electroporated with plasmid #3 (ChR2tc-mEos2), which sometimes showed unusual dendritic shapes and hot spots of fluorescence that may reflect protein aggregation. Targeted whole cell patch clamp recordings were performed from neurons expressing ChR2tc-GFP (*n* = 11 neurons in Dp) or ChR2wt-GFP-mbd (*n* = 2 neurons in the dorsal telencephalon) in an explant preparation of the whole brain (Zhu et al., 2012) at 7 dpe. All neurons had normal resting potentials between −60 and −75 mV. Whole-field light pulses (460 nm) of fixed intensity (250–300 μW/mm^2^) and different durations (1, 2, 5, 10, 20, 50 ms) were delivered at 1 Hz. In all neurons, action potentials were triggered reliably (probability > 90 %) when the duration of light pulses was 10 ms or longer (Figure 4A). Some neurons reliably fired action potentials even when light pulses were as short as 2 ms or 1 ms (not shown).

In order to examine synaptic transmission in Dp we expressed hEF1α::ChR2tc-GFP (plasmid #2) in Dp neurons by targeted IEP and prepared brain explant preparations at 7–10 dpe. Neurons were optically stimulated with trains of wide-field light pulses (460 nm; 10 ms duration, 10 pulses at 5 or 10 Hz). Whole cell voltage clamp recordings were performed from ChR2tc negative neurons in Dp that were usually intermingled with ChR2tc positive neurons (*n* = 42 neurons in 9 fish). Excitatory post-synaptic currents (EPSCs) and inhibitory post-synaptic currents (IPSCs) were measured by holding the recorded neurons close to the reversal potentials for chloride currents (−70 mV) and cation currents (0 mV), respectively. EPSCs time-locked to the optical stimulus were observed in only one neuron, and stimulus-locked IPSCs were observed in two neurons (Figures 4B,C). The EPSC and one of the IPSCs had short latencies (< 6 ms; Figures 4B,C), consistent with monosynaptic connections, while the second IPSC had a longer latency. These results demonstrate that monoysnaptic connectivity among Dp neurons is sparse. In addition, we observed slow inhibitory or excitatory currents that were not time-locked to the stimulus pulses in 23 of the 42 Dp neurons (Figure 4D). Together, these results show that electroporation can be used to introduce optogenetic probes into adult neurons to examine functional connectivity in the intact brain.

**Figure 5:**
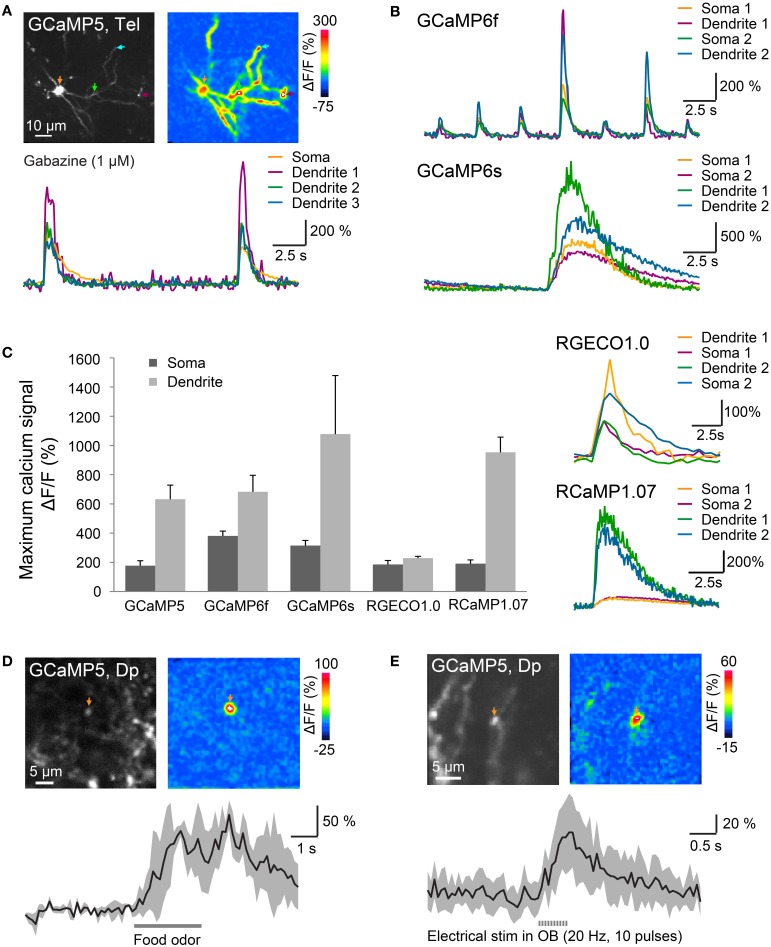
**Optical measurements of calcium signals after targeted electroporation of GECIs. (A)** Top left: expression of GCaMP5 in a neuron of the dorsal telencephalon after EEP of plasmid #11. Bottom: change in GCaMP5 fluorescence as a function of time. Colored traces correspond to the locations indicated by colored arrows in the images above. Large, low-frequency calcium transients were induced by Gabazine (1 μM), which causes epileptiform population activity. Top right: spatial distribution of fluorescence changes (ΔF/F) during a calcium transient, relative to baseline before the transient. **(B)** Calcium transients in the presence of Gabazine (1 μM) measured with GCaMP6f and GCaMP6s. **(C)** Mean amplitude (±SD) of calcium transients at the soma and at a dendritic location measured with different GECIs (*n* = 9 neurons from 3 fish for each GECI). Right: fluorescence transients of red calcium indicators in the presence of Gabazine. **(D)** Localized calcium transient in a Dp neuron expressing GCaMP5 (IEP of plasmid #6; 11 dpe), evoked by odor stimulation (food extract; average over 3 trials). **(E)** Localized calcium transients in a Dp neuron expressing GCaMP5 (IEP of plasmid #6; 8 dpe), evoked by electrical stimulation in the olfactory bulb (20 Hz, 10 pulses; average over 10 trials).

We next used EEP in the dorsal telencephalon to express different GECIs including the green-fluorescent probes GCaMP5 (Akerboom et al., 2012), GCaMP6f (fast variant of GCaMP6) (Chen et al., 2013), GCaMP6s (slow variant of GCaMP6) (Chen et al., 2013) and the red-fluorescent indicators RGECO1.0 (Zhao et al., 2011) and RCaMP1.07 (Ohkura et al., 2012) (Table 2, plasmids #11, #16, #17, #18, and #19; *n* = 3 or 4 fish for each GECI). At 7–10 dpe, fluorescence was examined by multiphoton microscopy in the *ex-vivo* preparation. In order to produce large changes in intracellular calcium concentration we applied the GABA_A_ receptor blocker Gabazine (1 μM) through the bath. This treatment is known to induce epileptiform bursting of many neurons in the forebrain at low inter-burst frequency (Tabor et al., 2008). Gabzine induced large changes in fluorescence intensity (ΔF/F) throughout the soma and dendrites of many GECI-expressing cells that occurred at frequencies of approximately 0.1–0.3 Hz (Figures 5A–C). The amplitude of these events was measured at the soma and at dendritic locations where the fluorescence in the time-averaged ΔF/F map was large. This procedure provides a simple assay to compare fluorescence changes of different GECIs produced by intense bursting of adult telencephalic neurons (Figure 5C).

Mean changes of GECI fluorescence in the presence of Gabazine were approximately 180–400% at somata and even larger in dendrites (Figure 5C; *n* = 9 neurons from 3 fish for each GECI). Particularly large fluorescence changes were observed with GCaMP6s (soma: approximately 300%; dendrite: approximately 1000%). Calcium transients observed with GCaMP6s (Figure 5B bottom) decayed more slowly than those produced by other GECIs, consistent with the slow kinetics of this probe (Chen et al., 2013). Substantial fluorescence transients were also observed with all other calcium sensors including the red-fluorescent indicators, RGECO1.0 and RCaMP1.07 (Figure 5C).

In Dp neurons expressing GCaMP5 (plasmid #6) by IEP we also measured fluorescence changes evoked by odor stimulation. Previous electrophysiological studies showed that many Dp neurons receive depolarizing synaptic input during odor stimulation but only a small subset fires action potentials (Yaksi et al., 2009; Blumhagen et al., 2011). In some Dp neurons, odor stimulation produced a calcium signal at the soma and a global calcium signal throughout the dendrite, indicative of action potential firing. In addition, we frequently observed smaller, highly localized calcium transients that most likely reflect subthreshold synaptic input in dendrites (Figure 5D). Similar calcium transients were also evoked by electrical stimulation (0.5 ms pulse duration, 10 pulses at 20 Hz) in the posterior olfactory bulb (Figure 5E). Together, these results show that various green- and red-fluorescent GECIs function efficiently in adult telencephalic neurons when they are introduced by electroporation.

## Discussion

We report methods to directly express transgenes in neurons of the adult zebrafish brain by HSV-1 or electroporation. Both methods are simple, efficient and can produce strong and long-lasting gene expression without obvious toxicity. In other species, fast gene transfer can be achieved using AAVs or lentiviruses but comparable methods have been lacking in zebrafish. This gap in the molecular toolbox for zebrafish may therefore be filled by HSV-1 and targeted electroporation.

HSV-1 is used for gene transfer in other species because it exhibits a high potential to infect neurons and low levels of toxicity. Our observations in zebrafish are fully consistent with these properties of HSV-1. However, we observed substantial variation in reporter gene expression between different HSV-1 viruses, presumably depending on the method of virus production and other factors. HSV-1 can produce dense expression of transgenes, which is important to target large populations of neurons. The ability of HSV-1 to retrogradely infect neurons via their axons can be exploited to target defined projection neurons. Conceivably, additional cell type selectivity may be generated by the choice of the promoter, which can be exchanged using established procedures (Simonato et al., 1999). We therefore expect that HSV-1 will become an important tool for gene transfer in zebrafish.

We established reliable protocols for gene transfer by electroporation using external or internal electrodes. Compared to gene transfer by HSV-1, transgene expression was sparser but strong. One advantage of electroporation is that plasmids can be introduced directly into neurons without the need to package genetic material into viruses or other vectors (Barnabé-Heider et al., 2008). The time to produce reagents for gene transfer and the risk of immune responses or other potential complications are therefore reduced. Moreover, the efficiency of electroporation should not vary substantially between cell types, brain areas and even species because electroporation relies on physical rather than molecular mechanisms. Electroporation is therefore a particularly fast and versatile method for gene transfer.

Electroporation has been used previously to introduce DNA into individual or small groups of neurons (Haas et al., 2001; Bianco et al., 2008; Kitamura et al., 2008) and to transfer transgenes into populations of cells near the ventricle (Barnabé-Heider et al., 2008). In order to target neuronal populations in specific brain regions we used internal electrodes (IEP) to create a local electrical field (Nishi et al., 1996). Physical damage was minimal because the electrodes were made of thin wires (25 μm) and the electrical pulses were adjusted to the local tissue parameters in each experiment. The procedure is not substantially more difficult to perform than viral injections and most likely applicable in different brain areas and species.

Efficient gene expression in the adult brain was achieved with a wide range of promoters that drive broad gene expression at early developmental stages. This result was not necessarily expected because gene expression in stable transgenic lines often becomes restricted in the adult brain (Stamatoyannopoulos et al., 1993; Goldman et al., 2001; Li et al., 2005; Zhu et al., 2009). A possible explanation for this result is that gene transfer into the adult brain bypasses silencing processes during development. Among the promoters tested, the heat-shock promoter zHsp70l appeared particularly promising to achieve broad, cell type-independent expression of transgenes.

We took advantage of electroporation to express a variety of ChR2 variants and GECIs in the adult telencephalon. Using a simple procedure to assess basic functional properties of GECIs in the intact brain we observed functional differences between GECIs that corresponded well to previous observations in other assays and species (Akerboom et al., 2012; Chen et al., 2013). For example, largest but also slowest fluorescence signals were observed with GCaMP6s (Chen et al., 2013). Consistent with previous results obtained in larvae (Walker et al., 2013) we observed substantial fluorescence signals using the red-fluorescent indicator RGECO1.0 (Zhao et al., 2011). Moreover, we obtained large fluorescence signals with another red-fluorescent GECI, RCaMP1.07 (Ohkura et al., 2012). These probes are therefore promising tools for multicolor calcium imaging.

Dp is a telencephalic brain area that is homologous to mammalian olfactory cortex (Mueller and Wullimann, 2009; Mueller et al., 2011) and assumed to be involved in olfactory memory (Wilson and Sullivan, 2011). Functional synaptic connectivity between principal neurons in olfactory cortex is difficult to analyze by paired electrophysiological recordings because it is very sparse (Johnson et al., 2000; Franks et al., 2011). This problem can be overcome by optogenetic stimulation of multiple neurons to increase the probability of finding a monosynaptically connected post-synaptic neuron (Franks et al., 2011). When ChR2tc was expressed in multiple Dp neurons by IEP we detected short-latency EPSCs or IPSCs in a small fraction of the recorded ChR2tc-negative neurons. Monosynaptic connectivity in Dp is therefore sparse, consistent with recurrent connectivity in mammalian olfactory cortex. Further experiments using this approach may now be performed to quantify connectivity in more detail. In Dp neurons expressing GECIs, odor stimulation evoked localized dendritic calcium transients that most likely reflect subthreshold synaptic input. Hence, sparse expression of GECIs by IEP is a promising approach to measure the tuning of synaptic inputs at different dendritic locations of Dp neurons. Conceptually similar experiments have provided insights into the processing of synaptic inputs in other brain areas such as the visual and auditory cortex (Jia et al., 2010; Chen et al., 2011). In summary, we conclude that gene transfer by HSV-1 and electroporation have a wide range of potential applications in zebrafish neuroscience. Moreover, electroporation is likely to be a useful technique for gene transfer in species in which genetic methods are not well established.

### Conflict of interest statement

The authors declare that the research was conducted in the absence of any commercial or financial relationships that could be construed as a potential conflict of interest.
